# The Burden of Pertussis Hospitalization in HIV-Exposed and HIV-Unexposed South African Infants

**DOI:** 10.1093/cid/ciw545

**Published:** 2016-11-02

**Authors:** Nasiha Soofie, Marta C. Nunes, Prudence Kgagudi, Nadia van Niekerk, Tselane Makgobo, Yasmeen Agosti, Cleopas Hwinya, Jayani Pathirana, Shabir A. Madhi

**Affiliations:** 1 Department of Science and Technology/National Research Foundation, Vaccine Preventable Diseases; 2 Medical Research Council, Respiratory and Meningeal Pathogens Research Unit, University of the Witwatersrand; 3 National Institute for Communicable Diseases, National Health Laboratory Service, Centre for Respiratory Diseases and Meningitis, Johannesburg, South Africa

**Keywords:** *Bordetella pertussis*, infants, incidence, HIV-exposed, neonates

## Abstract

***Background.*** There are limited data on pertussis in African children, including among human immunodeficiency virus (HIV)–exposed infants. We conducted population-based hospital surveillance to determine the incidence and clinical presentation of *Bordetella pertussis–*associated hospitalization in perinatal HIV-exposed and -unexposed infants.

***Methods.*** Children <12 months of age hospitalized with any sign or symptom of respiratory illness (including suspected sepsis or apnea in neonates) were enrolled from 1 January 2015 to 31 December 2015. Detailed clinical and demographic information was recorded and respiratory samples were tested by polymerase chain reaction (PCR).

***Results.*** The overall *B. pertussis* PCR positivity was 2.3% (42/1839), of which 86% (n = 36) occurred in infants <3 months of age. *Bordetella pertussis* was detected in 2.1% (n = 26/1257) of HIV-unexposed and 2.7% (n = 16/599) of HIV-exposed infants. The incidence (per 1000) of *B. pertussis*–associated hospitalization was 2.9 (95% confidence interval [CI], 1.8–4.5) and 1.9 (95% CI, 1.3–2.6) in HIV-exposed and HIV-unexposed infants, respectively (*P* = .09). The overall in-hospital case fatality ratio among the cases was 4.8% (2/42), both deaths of which occurred in HIV-exposed infants <3 months of age. Among cases, presence of cough ≥14 days (20.5%) and paroxysmal coughing spells (33.3%) at diagnosis were uncommon. Only 16 (38%) *B. pertussis–*associated hospitalizations fulfilled the Centers for Diseases Control and Prevention case definition of “definite” pertussis.

***Conclusions.*** *Bordetella pertussis* contributed to a modest proportion of all-cause respiratory illness hospitalization among black-African children, with a trend for higher incidence among HIV-exposed than HIV-unexposed infants. Maternal vaccination of pregnant women should be considered to reduce the burden of pertussis hospitalization in this population.

Despite whole-cell pertussis vaccine being developed since 1914 [1, 2], pertussis remains a leading vaccine-preventable cause of death globally [3]. In 2013, Liu et al estimated that 1% (n = 60 000) of under-5 childhood deaths globally were attributable to pertussis [4]. The burden of pertussis is, however, probably underrecognized, considering that whereas only 148 000 cases were reported to the World Health Organization (WHO) in 2008, in the same year an estimated 195 000 deaths were attributed to pertussis [5]. This may be due to limited availability of diagnostic assays for identifying pertussis illness in low- and middle-income countries and underrecognition of pertussis among young infants, who may not present with classical paroxysmal coughing spells [6] but nevertheless are at greatest risk for severe pertussis disease and death [7].

Recently, there has reportedly been a reemergence of pertussis in high-income countries [3, 8–12]. This could be due to more sensitive diagnostic tools such as molecular assays for detection of *Bordetella pertussis* rather than reliance on culture, heightened physician awareness of the disease, and lower threshold for investigating. Furthermore, the increase in pertussis is temporally associated with transitioning from whole-cell to acellular pertussis vaccine formulations in many high-income countries, with the duration of protection of the latter (approximately 5 years) being less than for whole-cell pertussis vaccine (4–12 years) [13–15].

There are limited data on the burden of severe pertussis-associated illness from sub-Saharan African countries since the mid-1990s [16, 17], and particularly in settings with a high prevalence of maternal HIV infection. A recent study conducted in Cape Town, South Africa, identified *B. pertussis* by polymerase chain reaction (PCR) in 7% of children <13 years of age hospitalized with lower respiratory tract infection (LRTI), with the median age of cases being 8 months. Furthermore, identification of *B. pertussis* trended toward being more common among HIV-infected (15.8%) and HIV-exposed but uninfected infants (10.9%) than HIV-unexposed infants (5.4%) [18]. The study was, however, not designed to quantify the incidence or risk ratio of pertussis-associated hospitalization between HIV-exposed and HIV-unexposed infants.

Furthermore, most burden-of-disease studies on pertussis selectively enrolled infants with predefined clinical symptoms such as prolonged cough, whereas neonates and infants might not necessarily present with the typical clinical profile of whooping cough as observed in the adult [19].

The aim of this study was to evaluate the incidence and clinical presentation of pertussis-associated hospitalization in HIV-exposed and -unexposed infants using broad inclusion criteria, in an African setting where acellular pertussis vaccine was introduced into the public immunization program since 2009.

## SUBJECTS AND METHODS

### Study Population

The study was conducted at Chris Hani Baragwanath Academic Hospital (CHBAH) in Soweto, South Africa. According to data provided by Statistics South Africa, the Soweto population includes approximately 25 628 infants <1 year of age over the 2015 calendar year, of whom we estimate approximately 90% would be hospitalized to the single public hospital in the area, CHBAH, in this community in which unemployment is high and only <10% of households have private medical insurance [20]. The study period extended from 1 January to 31 December 2015, and is ongoing during 2016. Active recruitment of participants occurred throughout the week. Any infant admitted at the discretion of the attending physicians who were independent of the study to the general medical unit at CHBAH who presented with signs or symptoms suggestive of a lower respiratory tract infection or neonatal sepsis was approached for participation in the study (Supplementary Table 1). Exclusion criteria included infants rehospitalized within 14 days of discharge from a previous admission, or whose parents were <18 years of age or from whom we could not ascertain consent.

Since April 2009, South Africa had transitioned from using a whole-cell pertussis vaccine to an acellular-containing pertussis vaccine (Pentaxim, Sanofi Pasteur, Lyon, France), which is scheduled to be given at 6, 10, and 14 weeks of age, with a booster dose at 15–18 months of age through the public immunization program.

### Sample Collection and Testing

Nasopharyngeal swabs were collected using a commercially available nylon flocked-tip swab (FLOQS, Copan Flock Technologies, Brescia, Italy). The swab was passed gently through the nostril toward the nasopharynx for a distance equal to that between the patient's nares and earlobe, rotated a few times, held in place for approximately 5 seconds, withdrawn, and placed in 2.5 mL of universal transport media (UTM; Copan Flock Technologies, Brescia, Italy). Nasopharyngeal aspirates were obtained by douching the nasopharynx with 2 mL of 0.9% sterile saline using a 5-mL syringe as described previously [21]. Mucus mixed with saline was then suctioned out and collected in a sterile container with UTM. Induced sputum collection took place in a well-ventilated room. Infants were given nothing by mouth for 4 hours prior to the procedure. The infants were nebulized with a bronchodilator to open and prepare the airways. Chest massages were conducted to induce the coughing up of sputum, which was then collected by suction into a sterile container. Specimens were transported on ice to the Respiratory and Meningeal Pathogens Research Unit laboratory (based at CHBAH). The comparison of yield for PCR testing using different methods are reported in the accompanying manuscript by Nunes et al in this supplement [22]. In this study, we included positivity for *B. pertussis* that was identified from any of the 3 sampling methods, although only nasopharyngeal swabs were undertaken on all subjects, the sensitivity of which was comparable (86%) to the composite yield from any of the 3 collection methods [22].

### Polymerase Chain Reaction Testing

The details on the methodology used for PCR testing are described in the manuscript by Nunes et al in this supplement [23]. In brief, samples were tested by real-time PCR for the presence of the multicopy pertussis insertion sequence (IS) *481* using a modified protocol to the one described by the Centers for Disease Control and Prevention (CDC) [24]. If IS*481* cycle threshold values were ≤40, total nucleic acids were reextracted and tested again for IS*481*, in a duplex reaction for hIS*1001* and pIS*1001* and in a singleplex reaction for the pertussis toxin subunit S1 (*ptxS1*). We followed the 4-target algorithm developed by Tatti et al in our second PCR for determining PCR positivity for *B. pertussis* [24]. Nasopharyngeal swab samples were also tested by PCR for respiratory syncytial virus (RSV), influenza virus, and human metapneumovirus (hMPV) using an in-house PCR as previously described [25]. The primers and probes (LTC Tech South Africa) used are listed in Supplementary Table 2.

### Clinical Information

Detailed clinical characteristics of symptoms classically associated with pertussis such as paroxysmal coughing with whoop, posttussive vomiting, apnea, cyanosis, and seizures was abstracted from the hospital medical records for each study participant. Information on additional signs and symptoms generally associated with a respiratory infection and intensive care unit admissions including ventilation history were also included. Based on the scoring system proposed by Preziosi et al to distinguish severe from nonsevere pertussis infection in the presence of a microbiologically confirmed *B. pertussis* infection in older patients, a Modified Preziosi Scale (MPS) was created to allow to stratify disease severity in infants [26]. A MPS score was calculated for each participant to measure severity of illness (Supplementary Table 4). A MPS score of ≤6 was classified as nonsevere, while that of ≥7 was considered a severe illness. Infants who tested *B. pertussis* positive were also screened using the CDC case definition for pertussis, which includes (1) acute cough illness of any duration with culture of *B. pertussis* from a clinical specimen, or (2) cough illness lasting ≥2 weeks with at least 1 of the following signs or symptoms: paroxysms of coughing, inspiratory whoop, posttussive vomiting, apnea (with or without cyanosis), and PCR positivity for pertussis [27]. Posthospital follow-ups of pertussis-associated cases for deaths were conducted via telephone interview. In utero HIV exposure was ascertained by review of the individual infant's Road to Health Chart (RTHC), record of laboratory tests, history of postbirth antiretroviral prophylaxis or treatment of the mother, and interview with the parent. Vaccination status against pertussis was ascertained from a review of the RTHC.

### Statistical Analyses

Age as a continuous or categorical variable was calculated for the overall enrolled infants and the *B. pertussis* PCR-positive infants. Categorical variables, including number of infants presenting with different symptoms, number who received antibiotics or were admitted to intensive care, and number categorized as severe or CDC confirmed or probable pertussis cases were described as proportions and compared by χ^2^ or Fisher exact test between HIV-exposed and HIV-unexposed infants. Continuous variables, including length of hospitalization and age at hospitalization, were represented as mean or median and compared by Student *t* test or Mann-Whitney test between HIV-exposed and HIV-unexposed infants. Logistic regression analysis was performed to assess associations between *B. pertussis* and select respiratory viral coinfections including RSV, hMPV, and influenza A/B virus.

**Figure 1.**
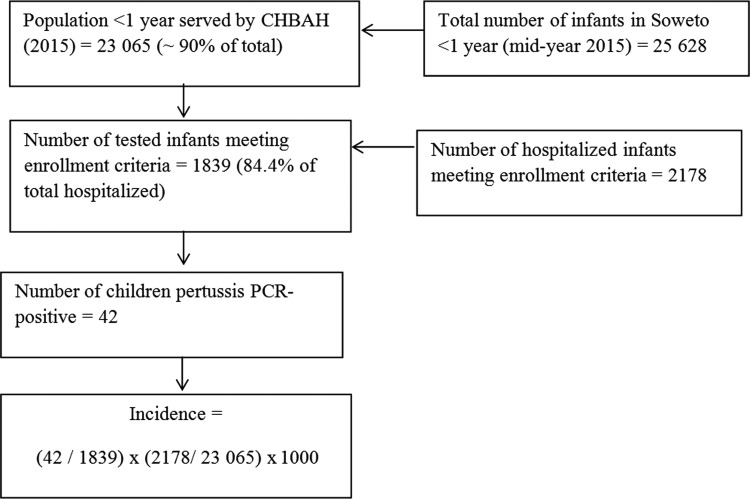
Flowchart of study enrollment at Chris Hani Baragwanath Academic Hospital (CHBAH) and methods for estimating population-based incidence of pertussis polymerase chain reaction (PCR)–positive associated hospitalizations and deaths overall. Incidence estimates were calculated separately for human immunodeficiency virus (HIV)–exposed and HIV-unexposed infants.

Using population denominator data available for Soweto (Johannesburg metropolitan area region D), incidence estimates were calculated. Incidence of pertussis-associated hospitalization was calculated per 1000 infants using the number of PCR-positive cases, adjusted for nonenrollments divided by the midyear total <1 year of age population estimate, multiplied by 1000 [20]. HIV prevalence in the study population was estimated from the projections of the Actuarial Society of South Africa AIDS and Demographic model; these estimates have been validated by comparison with other estimates and compare favorably with estimates from population-based HIV prevalence studies [28]. We estimated that 90% of the infants requiring hospitalization seek care at CHBAH. Also, we assume that the prevalence of pertussis cases in the nonenrolled infants was the same as in enrolled infants; the total population of children in Soweto <1 year of age obtained from the midyear estimates described each of the stratified age groups <1 year of age during the 12-month surveillance period (Figure 1). Specific denominators for HIV-exposed and HIV-unexposed infants were imputed based on 28% of pregnant women being infected in Soweto during the study period [29]. Pertussis-associated case fatality ratio was calculated based on the number of pertussis PCR-positive cases hospitalized at CHBAH who died during or within 2 weeks of hospitalization. *P* values <.05 were considered significant. Analyses were performed using Stata software, version 13.1 (StataCorp, College Station, Texas).

### Ethical Considerations

The study protocol was approved by the Human Research Ethics Committee of the University of the Witwatersrand (HREC number: 131109) and conducted in accordance with Good Clinical Practice guidelines. Legal guardians of the study participants were counseled on the study and study-related procedures prior to obtaining signed informed consent.

## RESULTS

A total of 2178 infants with symptoms of a lower respiratory tract infection (n = 1758, including 18 with apnea) or neonatal sepsis (n = 420) were hospitalized at CHBAH during 2015. Of these, 1865 (85.6%) infants were enrolled in the study, of whom 778 (41.8%) were female, 1788 (96.6%) of black-African descent, and 599 (32.3%) HIV exposed in utero; the median age at admission was 91 days (interquartile range [IQR], 33–192) (Table 1). Respiratory samples were collected from 1839 (98.6%) of the enrolled infants for *B. pertussis* PCR testing, all of whom had nasopharyngeal swabs except for 2 each who only had induced sputum and nasopharyngeal aspirate samples collected. Among infants for whom all 3 sample types were collected, the sensitivity for identifying *B. pertussis* from nasopharyngeal swab compared to the composite of detection by any of the 3 methods was 86%, as reported elsewhere [22]. We did not make any further adjustment for this observed sensitivity in our incidence calculations.

**Table 1.** Demographic Characteristics of the Infants Enrolled in the Study and Pertussis Polymerase Chain Reaction–Positive Infants

Characteristics	Infants Enrolled in the Study (N = 1865)	Infants Pertussis PCR-Positive (n = 42)
Age at hospitalization		
Age, d, median (IQR)	91 (33–192)	51 (42–73)
<3 mo	930 (49.9)	36 (85.7)
3 to <6 mo	422 (22.6)	4 (9.5)
6 to 12 mo	513 (27.5)	2 (4.8)
Race^a^		
Black-African	1788 (96.6)	38 (90.5)
White	59 (3.2)	4 (9.5)
Other	4 (0.2)	0
Female sex^b^	778/1861 (41.8)	26 (61.9)
HIV exposed^c^	599/1856 (32.3)	16 (38.1)

Data are presented as No. (%) unless otherwise indicated.

Abbreviations: HIV, human immunodeficiency virus; IQR, interquartile range; PCR, polymerase chain reaction.

^a^ Race unknown for 14 enrolled infants.

^b^ Sex unknown for 4 enrolled infants.

^c^ HIV exposure status unknown for 9 enrolled infants.

There were no differences in demographic characteristics between the 26 enrolled infants in whom testing for *B. pertussis* was not undertaken compared to the rest of the infants (Supplementary Table 3). Of the 1839 infants in whom *B. pertussis* PCR testing was undertaken, 42 (2.3%) tested positive. The median age at hospitalization for the *B. pertussis–*positive cases was 51 days (IQR, 42–73), and 36 (85.7%) were <3 months of age. Furthermore, of the pertussis-associated hospitalizations, 26 (61.9%) were female and 16 (38.1%) were HIV-exposed (Table 1). The detection rate of *B. pertussis* was similar among HIV-exposed (n = 16 [2.7%]) and HIV-unexposed infants (n = 26 [2.1%]) (*P* = .09).

### Clinical Presentation of the *B. pertussis–*Positive Cases

Forty-one of the 42 (95.1%) cases with *B. pertussis*–associated hospitalizations had a history of cough at admission, but only 8 (20.5%) reported a cough duration ≥14 days at enrollment. Furthermore, paroxysmal coughing spells were evident in 14 (33.3%) of the cases with *B. pertussis–*associated hospitalization. Twenty-eight (66.7%) of the cases were classified as severe illness using the MPS, while 16 (38.1%) fulfilled the CDC criteria of “confirmed cases” and 13 (30.1%) met the “probable pertussis” definition. Thirteen (30.1%) did not meet either the CDC “confirmed” or “probable” case definitions, including 1 participant who had no cough, other symptoms or culture attempted; 4 participants who had cough <2 weeks, no other symptoms, and tested negative on culture; 1 who had cough >2 weeks but no other symptoms and tested negative on culture; and 7 who had cough <2 weeks, no other symptoms, and tested negative on culture.

The mean length of the pertussis-associated hospitalization was 5.6 days, and antibiotics were prescribed during admission to 36 (85.7%) cases, although only 14 (33.3%) received a macrolide. Ten of the 14 (71.4%) infants who received a macrolide as empiric treatment had a history of cough for ≥14 days (n = 4) or presence of a paroxysmal cough (n = 6). Four (9.5%) infants, all <3 months of age (aged 4, 7, 7, and 8 weeks), were admitted to the intensive care unit, including 1 who required mechanical ventilation. Comparing the frequency of the clinical signs and symptoms associated with *B. pertussis* PCR positivity between HIV-exposed and HIV-unexposed infants, there were no differences (Table 2).

**Table 2.** Clinical Symptoms at Admission and Clinical Management of the Pertussis Polymerase Chain Reaction–Positive Infants by Human Immunodeficiency Virus Exposure Status

	All PCR-Positive Pertussis Cases	HIV-Exposed PCR-Positive Pertussis Cases	HIV-Unexposed PCR-Positive Pertussis Cases
Symptom	(n = 42)	(n = 16)	(n = 26)
Overall (age <12 mo)			
Symptoms during hospitalization			
Cough	41 (95.1)	16 (100)	25 (96.2)
Apnea	4 (11.1)	1 (7.7)	3 (13.0)
Paroxysmal cough with whoop	3 (7.1)	1 (6.3)	2 (7.7)
Paroxysmal cough without whoop	11 (26.2)	5 (31.3)	6 (23.1)
Pulmonary signs	41 (97.6)	16 (100)	25 (96.2)
Seizure	2 (5.1)	1 (7.1)	1 (4.0)
Tachypnea	19 (50.0)	8 (50.0)	11 (50.0)
Chest wall retraction	25 (59.5)	11 (68.8)	14 (53.9)
Cyanosis	2 (4.9)	0	2 (7.7)
Conjunctival injections	1 (2.7)	0	1 (4.2)
Vomiting	7 (16.7)	2 (12.5)	5 (19.2)
Coryza	7 (18.4)	2 (13.3)	5 (21.7)
Fever	5 (13.2)	2 (14.3)	3 (12.5)
Movement only when stimulated	4 (10.5)	2 (13.3)	2 (8.7)
Poor feeding	10 (23.8)	5 (31.3)	5 (19.2)
Wheezing on auscultation	7 (17.1)	2 (12.5)	5 (20.0)
Cough duration ≥14 d	8 (20.5)	3 (18.8)	5 (21.7)
Antibiotic treatment during hospitalization	36 (85.7)	16 (100)	20 (79.9)
Macrolides	14 (33.3)	3 (18.8)	11 (42.3)
Admissions to intensive care	4 (9.5)	2 (12.5)	2 (7.7)
Age at hospitalization, d, median (IQR)	51 (42–73)	57 (41–87)	51 (42–73)
Cases with MPS score ≥7 (severe)	28 (66.7)	11 (68.8)	17 (65.4)
Length of hospitalization, d, mean (SD)	5.6 (4.9)	5.8 (5.6)	5.5 (4.6)
Fulfilling CDC criteria for confirmed pertussis	16 (38.1)	8 (50.0)	8 (30.8)
Fulfilling CDC criteria for probable pertussis (excluding confirmed)	13 (31.0)	5 (31.3)	8 (30.8)
	(n = 36)	(n = 13)	(n = 23)
<3 mo old			
Symptoms during hospitalization			
Cough	35 (97.2)	13 (100)	22 (95.7)
Apnea	4 (13.3)	1 (10.1)	3 (15.0)
Paroxysmal cough with whoop	3 (8.3)	1 (7.7)	2 (8.7)
Paroxysmal cough without whoop	10 (27.8)	4 (30.8)	6 (26.1)
Pulmonary signs	35 (97.2)	13 (100)	22 (95.7)
Seizure	2 (6.1)	1 (9.1)	1 (4.6)
Tachypnea	15 (48.5)	6 (46.2)	9 (47.4)
Chest wall retraction	20 (55.6)	8 (61.5)	12 (52.2)
Cyanosis	2 (5.7)	0	2 (8.7)
Conjunctival injections	1 (3.2)	0	1 (4.8)
Vomiting	7 (19.4)	2 (15.4)	5 (21.7)
Coryza	7 (21.9)	2 (16.7)	5 (25.0)
Fever	4 (12.1)	1 (8.3)	3 (14.3)
Movement only when stimulated	3 (9.4)	1 (8.3)	2 (10.0)
Poor feeding	9 (25.0)	4 (30.8)	5 (21.7)
Wheezing on auscultation	5 (14.3)	2 (15.4)	3 (13.6)
Cough duration ≥14 d	7 (21.2)	3 (23.1)	4 (20.0)
Antibiotic treatment during hospitalization	32 (88.9)	13 (100)	19 (82.6)
Macrolides	14 (38.9)	3 (23.1)	11 (47.8)
Admission to intensive care	4 (11.1)	2 (15.4)	2 (8.7)
Age at hospitalization, d, median (IQR)	49 (35–64)	49 (35–65)	50 (35–59)
Cases with MPS score ≥7 (severe)	24 (66.7)	9 (69.2)	15 (65.2)
Length of hospitalization, d, mean (SD)	6.1 (5.1)	6.5 (6.0)	6.0 (4.7)
Fulfilling CDC criteria for confirmed pertussis	15 (41.7)	7 (53.9)	8 (34.8)
Fulfilling CDC criteria for probable pertussis (excluding confirmed)	12 (33.3)	4 (30.8)	8 (34.8)

Data are presented as No. (%) unless otherwise indicated.

Abbreviations: CDC, Centers for Disease Control and Prevention; HIV, human immunodeficiency virus; IQR, interquartile range; MPS, Modified Preziosi Scale; PCR, polymerase chain reaction; SD, standard deviation.

Among the 42 subjects with *B. pertussis*–associated hospitalizations, 9 (21.4%) were coinfected with 1 of the 3 tested respiratory viruses, including 7 with RSV and 2 with hMPV. The prevalence of identifying any of these respiratory viral infections was similar among the *B. pertussis* PCR-positive and the PCR-negative infants for RSV (16.7% vs 26.1%; *P* = .17), hMPV (4.8% vs 2.2%; *P* = .27), and influenza virus (0% vs 3.2%; *P* = .64) (Table 3). The clinical course of *B. pertussis–*associated hospitalization did not differ between those with and those without the presence of a respiratory virus (data not shown).

**Table 3.** Association of Respiratory Viruses and Pertussis Illness

Virus	Pertussis PCR-Positive Cases	Non-Pertussis PCR-Positive Cases	OR (95% CI)	*P* Value
RSV	7/42 (16.7)	471/1797 (26.2)	0.6 (.2–1.3)	.17
Influenza	0/42 (0)	57/1797 (3.2)	…	.64*
Human metapneumovirus	2/42 (4.8)	39/1797 (2.2)	2.3 (.5–9.7)	.27
At least 1 respiratory virus	9/42 (21.4)	564/1797 (31.4)	0.6 (.3–1.3)	.17

Data are presented as No. (%) unless otherwise indicated.

Abbreviations: CI, confidence interval; OR, odds ratio calculated by regression analysis; PCR, polymerase chain reaction; RSV, respiratory syncytial virus.

*P value calculated by Fisher exact test.

The overall case fatality ratio of pertussis-associated hospitalizations was 4.8% (2/42). Both deaths occurred in HIV-exposed females (5 and 9 weeks of age) who were born preterm at 32 and 34 weeks of gestational age, and neither of whom received any dose of pertussis-containing vaccine. The first infant was admitted at 35 days of age and died 14 days later. She had presented with a 3-day history of cough, posttussive vomiting, and coryza. The lymphocyte count at admission was 8.9 × 10^9^ cells/L and lymphocyte percentage was 75.3%, and RSV was also detected by PCR in the induced sputum specimen. The infant was diagnosed by the attending physician as having bronchopneumonia and treated with cefotaxime. The second infant was 79 days of age at admission and presented with a 4-day history of cough, respiratory rate of 60 breaths per minute, lower chest wall indrawing, lymphocyte count of 16.5 × 10^9^ cells/L, and lymphocyte percentage of 60.3%. This infant was HIV infected and was diagnosed as having an unspecified acute LRTI and was treated with cefotaxime. The infant died 15 days following admission. Neither infant was treated empirically with a macrolide by the attending physicians.

### Incidence of Pertussis-Associated Hospitalizations

Applying the algorithm shown in Figure 1, the overall incidence of *B. pertussis*–associated hospitalizations in infants was 2.2 (95% confidence interval [CI], 1.6–2.8) cases per 1000 infants. Restricting the analysis to infants <3 months of age, the incidence was 1.8 (95% CI, 1.3–2.4) cases per 1000 infants compared with 0.3 (95% CI, .1–.6) cases per 1000 infants for the 3- to <12-month age group (risk ratio, 5.9 [95% CI, 2.8–12.4]) (Table 4). The estimated incidence of pertussis-associated hospitalizations was slightly higher in HIV-exposed compared to HIV-unexposed infants; however, these differences were nonsignificant across all age groups. The imputed incidence of pertussis deaths in infants <6 months of age was 10 (95% CI, 2–30) deaths per 100 000 infants.

**Table 4.** Population-Based Incidence of Pertussis Polymerase Chain Reactive–Positive Associated Hospitalizations and Deaths

Age Group	All Pertussis PCR-Positive Cases		HIV-Exposed Pertussis PCR-Positive Cases		HIV-Unexposed Pertussis PCR-Positive Cases	
	No. of Cases	Cases/1000 Infants^a^ (95% CI)	No. of Cases	Cases/1000 Infants^a^ (95% CI)	No. of Cases	Cases/1000 Infants^a^ (95% CI)
All pertussis PCR-positive cases						
Overall	42	2.2 (1.6–2.8)	16	2.9 (1.8–4.5)	26	1.9 (1.3–2.6)
<3 mo old	36	1.8 (1.3–2.4)	13	2.4 (1.4–3.8)	23	1.6 (1.1–2.3)
3 to <6 mo old	4	0.2 (.06–.5)	1	0.2 (.004–.8)	3	0.2 (.06–.6)
<6 mo old	40	2.1 (1.5–2.7)	14	2.6 (1.5–4.0)	26	1.9 (1.3–2.6)
6 to <12 mo old	2	0.1 (.02–.3)	2	0.4 (.09–1.2)	0	…
Outcome of death <6 mo old	2	0.1 (.02–.3)	2	0.4 (.09–1.2)	0	…
Severe pertussis PCR-positive cases (MPS score ≥7)						
Overall	28	1.4 (1.0–2.0)	11	2.0 (1.1–3.3)	17	1.2 (.7–1.8)
<3 mo old	24	1.2 (.9–1.8)	9	1.7 (.9–2.9)	15	1.1 (.7–1.7)
3 to <6 mo old	2	0.1 (.02–.3)	0	…	2	0.1 (.03–.5)
<6 mo old	26	1.3 (.9–1.9)	9	1.7 (.9–2.9)	17	1.2 (.7–1.8)
6 to <12 mo old	2	0.1 (.02–.3)	2	0.4 (.09–1.2)	0	…
Outcome of death <6 mo old	2	0.1 (.02–.3)	2	0.4 (.09–1.2)	0	…
Nonsevere pertussis PCR-positive (MPS ≤6)						
Overall	14	0.7 (.4–1.1)	5	0.9 (.4–2.0)	9	0.6 (.3–1.1)
<3 mo old	12	0.6 (.4–1.0)	4	0.7 (.2–1.6)	8	0.6 (.3–1.1)
3 to <6 mo old	2	0.1 (.02–.3)	1	0.2 (.004–.8)	1	0.1 (.001–.3)
<6 mo old	14	0.7 (.4–1.1)	4	0.7 (.2–1.6)	9	0.6 (.3–1.1)
6 to <12 mo old	0	…	0	…	0	…

There were 2 deaths in infants aged <6 months (35 days old and 64 days old), and zero deaths in children aged 6 to <12 months. There was no difference in incidences between HIV-exposed and HIV-unexposed infants. The analysis assumed that (1) 90% of infants with pertussis requiring hospitalization came to the study facility; (2) 84% of the eligible infants were tested for pertussis; (3) the prevalence of pertussis in the nontested infants was the same as in the tested infants; and (4) the proportion of HIV-exposed infants in the eligible nonenrolled infants was the same as in the tested infants.

Abbreviations: CI, confidence interval; HIV, human immunodeficiency virus; MPS, Modified Preziosi Scale; PCR, polymerase chain reaction.

^a^ Same as annual incidence.

Stratifying the severity of the pertussis PCR-positive cases according to the MPS, 28 cases were classified as severe, resulting in an incidence of 1.4 (95% CI, 1.0–2.0) cases per 1000 infants, whereas the incidence of nonsevere cases was 0.7 (95% CI, .4–1.1) cases per 1000 infants (Table 4). Both infants who died had an MPS score ≥7, as did the infant who required mechanical ventilation.

### Immunization History of the Pertussis PCR-Positive Infants

Eleven (26%) cases were too young (<6 weeks of age) to have received any pertussis-containing vaccine at the time of their pertussis-associated hospitalization. Of the remaining 31 cases, immunization records were available for 24 (77.4%). Of the 2 cases eligible to have received 2 vaccine doses, both received 2 doses but the second dose was received <14 days before the onset of the pertussis-associated hospitalization. Of the 5 cases old enough to receive 3 vaccine doses, only 1 was fully vaccinated (Table 5).

**Table 5.** Immunization Status of the Pertussis Polymerase Chain Reaction–Positive Infants

Age	Total Cases	Information Available	DTaP Dose 1 (6 wk)	DTaP Dose 2 (10 wk)	DTaP Dose 3(14 wk)
0–6 wk	11	…	0	0	0
7–10 wk	21	17	11^a^	0	0
11–14 wk	4	2	2	2^b^	0
>14 wk	6	5	5	4^c^	2^d^

Data are presented as No.; immunization status was ascertained by review of the immunization cards.

Abbreviation: DTaP, pentavalent vaccine containing diphtheria toxoid, tetanus toxoid, acellular pertussis, trivalent inactivated polio vaccine, and *Haemophilus influenzae* type b conjugate vaccine.

^a^ Seven pertussis admissions ≤14 days postvaccination.

^b^ Two pertussis admissions ≤14 days postvaccination.

^c^ One pertussis admission ≤14 days postvaccination.

^d^ One pertussis case missed the 10-week dose.

## DISCUSSION

The high burden of *B. pertussis–*associated hospitalization reported in this study (2.2 per 1000 infants) supplements the data from an earlier longitudinal cohort study undertaken in the same community in 2011 [23]. In the latter study, which included active weekly follow-up of infants for any respiratory illness and which was undertaken 3 years prior to this study, we documented an overall incidence of 29.2 and 37.2 per 1000 HIV-unexposed and HIV-exposed infants, respectively, during the first 6 months of life [23]. Furthermore, only 6 of the 37 (16.2%) *B. pertussis–*associated illnesses in the latter study resulted in hospitalization. Although the seasonality of *B. pertussis* circulation precludes any direct comparisons being made between these separate studies, these results together nevertheless suggest a high incidence of *B. pertussis–*associated illness in our setting. Data such as these are largely missing from low- and middle-income countries, impeding informing policy on whether maternal pertussis vaccination warrant implementation in settings such as ours. Furthermore, our study suggested a nonsignificantly higher incidence of pertussis-associated hospitalization in our setting in HIV-exposed infants, which is similar to what we had observed previously for invasive group B *Streptococcus* disease and RSV hospitalization in the first 6 months of life [30, 31]. This higher burden of pertussis-associated hospitalization among HIV-exposed infants, among whom the majority (86%) of cases occurred by 3 months of age, is likely due to lower transplacental acquisition of pertussis-specific maternal antibody in fetuses born to HIV-infected compared with HIV-uninfected women [32].

The incidence of pertussis-associated hospitalization identified by us is similar to the incidence of pertussis reported in the United States in 2014 (1.7/1000 infants) in the <6-month age group [33]. Similarly, Cortese et al, using nationally representative hospitalization discharge databases, estimated that the incidence of pertussis hospitalization in infants <12 months of age in 2003 was 65 (95% CI, 59–72) per 100 000 live births. In the same study, infants 1–2 months of age had the highest incidence (239 hospitalizations per 100 000 live births) and constituted 95% of pertussis cases that required mechanical ventilation; 100% of those who died were <3 months of age [34]. The incidence reported by Cortese et al was 2-fold greater than identified from a passive reporting system, supporting the notion that relying on passive reporting would underestimate the burden of pertussis hospitalization even in the United States [35, 36].

Although studies on the burden of pertussis from African countries are limited, a cross-sectional study from Uganda among 449 children aged 2 months to 12 years who presented with cough ≥14 days at a national referral hospital reported a prevalence of 15% (95% CI, 12%–18%) detected by an in-house PCR assay. The prevalence of *B. pertussis* identification was 3.0-fold (95% CI, 1.1- to 8.3-fold) greater among infants <23 months of age than children aged 24–59 months [12]. In contrast, a study conducted over 5 months in a national hospital in Niger that enrolled children <5 years of age with clinical suspicion of pertussis reported a prevalence of 0.82% for identifying *Bordetella* species [37].

More recently, a study from Cape Town, South Africa, reported a 7% prevalence for detection of *B. pertussis* in children hospitalized with LRTI, with the median age of pertussis-associated cases being 8 months. However, only 41 (9%) of infants in that study were <2 months of age, of whom only 6 (1.3%) tested positive for *B. pertussis*. This study used a dual-target commercial kit (LightMix) for PCR detection instead of an in-house PCR protocol [18]. The multiple-target in-house PCR used by us, and the evaluation of the cycle threshold values for both IS*481* and *ptxS1* targets to determine positivity with high specificity following the published CDC protocol, might have contributed in part to the differences observed in prevalence of pertussis-associated LRTI hospitalization between this study and that reported by us. This could have been further confounded by the different years during which these studies were performed and differences in inclusion criteria for testing for pertussis between the studies [24]. In this supplement, also reported on is the prevalence of *B. pertussis* identification in a separate multicountry study that was undertaken by the Pneumonia Etiology Research for Child Health (PERCH) group, which included investigating for *B. pertussis* among children hospitalized with WHO-defined severe and very severe pneumonia from August 2012 to January 2014 [38]. Included among the 7 PERCH sites was our site from Soweto (South Africa), which reported the highest positivity for *B. pertussis* in the 1- to 5-month age group (4% vs an overall prevalence of 1.2% across all the sites). The lack of standardization in enrollment criteria, the different periods of time when these studies were conducted, and differences in age group and laboratory assays, however, generally limit any direct comparisons from being made between these studies.

Consistent with the published literature, we observed that 76% of the pertussis PCR-positive infants at the time of hospitalization were too young (<10 weeks of age) to have received at least 1 dose of a pertussis-containing vaccine, and only 1 infant was fully vaccinated with the primary series of 3 doses [18, 39, 40]. Recent studies suggest that vaccination with acellular pertussis during pregnancy is safe and effective at protecting infants from pertussis, and that it may also impact the mortality in infants too young to be vaccinated [41]. It is unclear whether maternal vaccination would have affected the risk of disease or outcome in these children, among whom transplacental acquisition of maternal antibody could have been reduced both due to underlying HIV infection in the mothers as well as the preterm birth of the babies [23]. Whether maternal immunization would be able to prevent diseases in infants born to HIV-infected women requires further study.

In this study, we used a low threshold for investigating *B. pertussis–*associated hospitalization, primarily to address whether there was a spectrum of nonclassical pertussis-like illness that was being missed. Our study confirmed that *B. pertussis* cases among neonates and infants generally do not present with the classical signs and symptoms of whooping cough, which occurs mainly among older children. In addition, applying the current CDC case definition of pertussis to our study population, only 38% of the pertussis PCR-positive cases fulfilled the CDC criteria of “confirmed” pertussis. This calls to attention the different definitions used to define the disease from a clinical perspective and to determine the true epidemiologic burden of pertussis if relying solely on clinical presentation or requiring a culture-positive result. The increased use of PCR as a diagnosis tool will help to provide new definitions of clinical disease, and future studies are needed to validate and determine the accuracy of these definitions. Further, an assessment of case severity according to the MPS showed that the majority (67%) of the pertussis PCR-positive cases were classified as severe cases; however, this is not surprising as all the infants were hospitalized, and it would be useful to employ this scoring scale in studies not restricted to hospitalized infants.

Limitations of our study included it being conducted over a single year, which could influence the incidence results, as *B. pertussis* circulation displays seasonal and temporal periodicity. Nevertheless, previous studies from the same setting in 2011 [23], and subsequently in 2012–2014 [38], persistently identified a role of *B. pertussis* in all-severity respiratory illness and severe pneumonia. A further limitation of our study was that surveillance was performed only at a single hospital, which limits the generalizability of our findings, as is also the case for most other studies from Africa.

In conclusion, although *B. pertussis* was only identified in a modest percentage of investigated cases in our study, the incidence of pertussis-associated hospitalization reported by us was as high as that reported in the United States [33]. Furthermore, similar to elsewhere, the incidence was greatest in infants <3 months of age, who also accounted for the majority of severe cases. Further studies are required to delineate whether in utero HIV exposure predisposes to increased susceptibility to *B. pertussis–*associated hospitalizations in settings such as ours, as suggested by our study. Similarly, the role of vaccination of pregnant women against pertussis, aimed at protecting their infants, warrants evaluation in HIV-infected and HIV-uninfected women in settings such as ours.

## Supplementary Data

Supplementary materials are available at http://cid.oxfordjournals.org. Consisting of data provided by the author to benefit the reader, the posted materials are not copyedited and are the sole responsibility of the author, so questions or comments should be addressed to the author.

Supplementary Data
