# Supplementary material for: The Burden of Pertussis Hospitalization in HIV-Exposed and HIV-Unexposed South African Infants
Source: Clin Infect Dis. 2016 Nov 2;63(Suppl 4):S165–73. doi: 10.1093/cid/ciw545 (PMC5106620; doi:10.1093/cid/ciw545)
Supplement: Supplementary Data [file supp_63_suppl-4_S165__index.html]

Supplementary Data 

# The Burden of Pertussis Hospitalization in HIV-Exposed and HIV-Unexposed South African Infants

## Supplementary Data

Supplementary Data

- Supplementary Data - Docx file
