# Supplementary material for: The Burden of Pertussis Hospitalization in HIV-Exposed and HIV-Unexposed South African Infants
Source: Clin Infect Dis. 2016 Nov 2;63(Suppl 4):S165–73. doi: 10.1093/cid/ciw545 (PMC5106620; doi:10.1093/cid/ciw545)
Supplement: Supplementary Data [file supp_ciw545_ciw545supp.docx]

Supplementary Table 1. Clinical presentation criteria for enrolment of hospitalized children

| Severe acute respiratory infection  (<3 months old) | Any child with diagnosis of suspected sepsis OR  Physician diagnosed LRTI irrespective of signs and symptoms OR  Apnoea |
| --- | --- |
| Physician diagnosed LRTI  (≥3 months old to <12 months) | Any child with physician-diagnosed LRTI including bronchiolitis, pneumonia, bronchitis and pleural effusion. |
| Severe acute respiratory infection  (≥3 months old to <12 months) | Any child with cough or difficult breathing AND  Any general danger sign* OR  Chest indrawing or stridor in a calm child OR  Tachypnoea |

*General danger signs are: unable to drink or breast-feed, vomits everything, convulsions, lethargy or unconsciousness.

Supplementary Table 2. Primers and Probes sequences

| Gene Name or organism | Sequence (5’-3’) |
| --- | --- |
| *IS481* | For 5’ CAAGGCCGAACGCTTCAT 3’  Rev 5’ GAGTTCTGGTAGGTGTGAGCGTAA 3’  Probe 5’ NED-CAGTCGGCCTTGCGTGAGTGGG-MGB 3’ |
| *PtxS* | For 5’ CGCCAGCTCGTACTTC 3’  Rev 5’ GATACGGCCGGCATT 3’  Probe 5’ VIC-AATACGTCGACACTTATGGCGA-MGB-3’ |
| *hIS1001* | For 5’ GGCGACAGCGAGACAGAATC 3’ |
|  | Rev 5’ GCCGCCTTGGCTCACTT 3’ |
|  | Probe 5’ VIC-CGTGCAGATAGGCTTTTAGCTTGAGCGC-MGB 3’ |
| *pIS1001* | For 5’ TCGAACGCGTGGAATGG 3’ |
|  | Rev 5’ GGCCGTTGGCTTCAAATAGA -3’ |
|  | Probe 5’ FAM-AGACCCAGGGCGCACGCTGTC-MGB 3’ |
|  | For 5’ AGATTTGGACCTGCGAGCG 3’ |
| Human *rnaseP* | Rev 5’ GAGCGGCTGTCTCCACAAGT-3’ |
|  | Probe 5’ FAM-TTCTGACCTGAAGGCTCTGCGCG-MGB 3’ |
|  | For 5’ GCTCCTCCTGTTCGACAGTCA 3’ |
| Human *GAPDH* | Rev 5’ ACCTTCCCCATGGTGTCTGA 3’ |
|  | Probe 5’ NED-CGTCGCCAGCCGAGCCACA-MGB 3’ |
|  | For 5’ AGATCAACTTCTGTCATCCAGCAA 3’ |
| RSV-A | Rev 5’ TTCTGCACATCATAATTAGGAGTATCAAT 3’ |
|  | Probe 5’ FAM-CACCATCCAACGGAGCACAGGAGAT-MGB 3’ |
|  | For 5’ GATGGCTCTTAGCAAAGTCAAGTTAA 3’ |
| RSV-B | Rev 5’ TGTCAATATTATCTCCTGTACTACGTTGAA 3’ |
|  | Probe 5’ VIC-ATACATTAAATAAGGATCAGCTGCTGTCATCCA-MGB 3’ |
|  | For 5’ GACCRATCCTGTCACCTCTGAC 3’ |
| Influenza-A | Rev 5’ AGG GCATTYTGGACAAAKCGT 3’ |
|  | Probe 5’ FAM-TGCAGTCCTCGCTCACTGGGCACG-MGB 3’ |
|  | For 5’ AAATACGGTGGATTAAAYAAAAGCAA 3’ |
| Influenza-B | Rev 5’ CCAGCAATAGCTCCGAAGAAA 3’ |
|  | Probe 5’ VIC-CACCCATATTGGGCAATTTCCTATGGC-MGB 3' |
|  | For 5’ GAAGARATAGACAAAGARGCAAG 3’ |
| hMPV | Rev 5’ TCCCACTTCTATRGTTGATGCTAG 3’ |
|  | Probe 5’ NED-TCAGCACCAGACACACC-MGB 3’ |

## Supplementary Table 3. Demographic characteristics of the infants tested and not tested for pertussis by PCR

| Characteristics | Infants tested by PCR  N=1839 | Infants not tested by PCR  N=26 | p-value |
| --- | --- | --- | --- |
| Age at hospitalization |  |  |  |
| Median age in days (IQR) | 91 (33, 193) | 75 (28, 125) | 0.11 |
| N. infants <3months (%) | 915 (49.8) | 15 (57.7) |  |
| N. infants 3-<6 months (%) | 415 (22.6) | 7 (26.9) | 0.38 |
| N. infants 6-12 months (%) | 509 (27.7) | 4 (15.4) |  |
| Race |  |  |  |
| N. Black-African (%) | 1762 (96.6) | 26 (100) |  |
| N. Caucasian (%) | 59 (3.2) | 0 | 0.99 |
| N. Other (%) | 4 (0.2) | 0 |  |
| N. female (%) | 766/1835 (41.7) | 12 (46.2) | 0.65 |
| N. HIV-exposed (%) | 589/1830 (32.2) | 10 (38.5) | 0.48 |

Race unknown for 14 infants.

Gender unknown for 4 enrolled infants.

HIV-exposure status unknown for 9 infants.

IQR: Interquartile range.

Supplementary Table 4. Modified Preziosi Scale scoring system

**Severe Pertussis Disease**: score ≥ 7 points on the MPS.

**Non-Severe Pertussis Disease**: score 1-6 points on the MPS
